# Androgen Effects on the Adrenergic System of the Vascular, Airway, and Cardiac Myocytes and Their Relevance in Pathological Processes

**DOI:** 10.1155/2020/8849641

**Published:** 2020-11-12

**Authors:** Abril Carbajal-García, Jorge Reyes-García, Luis M. Montaño

**Affiliations:** Departamento de Farmacología, Facultad de Medicina, Universidad Nacional Autónoma de México, CDMX, Mexico

## Abstract

**Introduction:**

Androgen signaling comprises nongenomic and genomic pathways. Nongenomic actions are not related to the binding of the androgen receptor (AR) and occur rapidly. The genomic effects implicate the binding to a cytosolic AR, leading to protein synthesis. Both events are independent of each other. Genomic effects have been associated with different pathologies such as vascular ischemia, hypertension, asthma, and cardiovascular diseases. Catecholamines play a crucial role in regulating vascular smooth muscle (VSM), airway smooth muscle (ASM), and cardiac muscle (CM) function and tone.

**Objective:**

The aim of this review is an updated analysis of the role of androgens in the adrenergic system of vascular, airway, and cardiac myocytes. *Body.* Testosterone (T) favors vasoconstriction, and its concentration fluctuation during life stages can affect the vascular tone and might contribute to the development of hypertension. In the VSM, T increases *α*1-adrenergic receptors (*α*_1_-ARs) and decreases adenylyl cyclase expression, favoring high blood pressure and hypertension. Androgens have also been associated with asthma. During puberty, girls are more susceptible to present asthma symptoms than boys because of the increment in the plasmatic concentrations of T in young men. In the ASM, *β*_2_-ARs are responsible for the bronchodilator effect, and T augments the expression of *β*_2_-ARs evoking an increase in the relaxing response to salbutamol. The levels of T are also associated with an increment in atherosclerosis and cardiovascular risk. In the CM, activation of *α*_1A_-ARs and *β*_2_-ARs increases the ionotropic activity, leading to the development of contraction, and T upregulates the expression of both receptors and improves the myocardial performance.

**Conclusions:**

Androgens play an essential role in the adrenergic system of vascular, airway, and cardiac myocytes, favoring either a state of health or disease. While the use of androgens as a therapeutic tool for treating asthma symptoms or heart disease is proposed, the vascular system is warmly affected.

## 1. Introduction

### 1.1. Metabolic Pathways of Steroids

**Figure 1:**
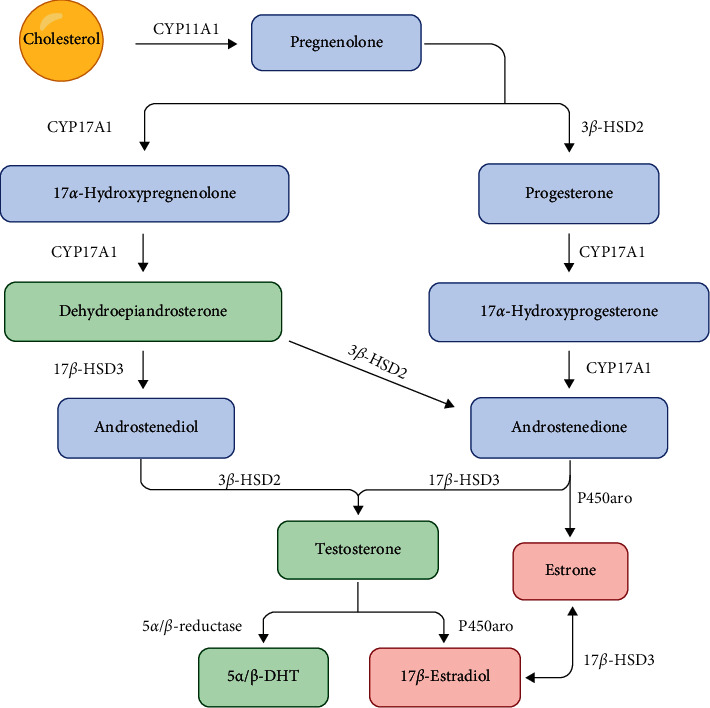
Androgen synthesis from cholesterol. Steroidogenesis in males is carried out mainly by Leydig cells and in females by theca and granulosa cells. Cholesterol is the precursor of all sex steroids, and its conversion to pregnenolone is mediated by the cholesterol side-chain cleavage cytochrome P450 enzyme (CYP11A1/P450scc). Once formed, this progestogen is converted into progesterone by 3*β*-hydroxysteroid dehydrogenase (3*β*-HSD2). Then, 17*α*-hydroxylase/17,20 lyase (CYP17A1/P450c17) hydroxylates pregnenolone to produce 17*α*-hydroxypregnenolone and subsequently removes the acetyl group to form dehydroepiandrosterone (DHEA). This last product can be either converted into androstenedione via 3*β*-HSD2 or into androstenediol by 17*β*-hydroxysteroid dehydrogenase (17*β*-HSD3). Androstenedione and androstenediol are further biotransformed to testosterone by 17*β*-HSD3 and 3*β*-HSD2, respectively. Furthermore, testosterone can be reduced to 5*α*- or 5*β*-dihydrotestosterone (5-*α*/*β*-DHT) by 5-*α*/*β*-reductases. Furthermore, P450 aromatase (P450aro) may convert testosterone into 17*β*-estradiol and androstenedione into estrone. Finally, 17*β*-HSD3 catalyzes the formation of 17*β*-estradiol from estrone.

Testosterone (T), the main testicular hormone, is produced by Leydig cells in high concentrations (95%). However, smaller amounts of T are also synthesized by the adrenal cortex [1–4]. The production and secretion of this androgen are regulated through luteinizing hormone (LH) stimulation. Cholesterol is the precursor of T, and the steroidogenesis is carried out through cytochrome P450 enzymes [5]. The conversion of cholesterol to pregnenolone is the first step in producing T and is accomplished by the P450 side-chain cleavage enzyme (P450cc/CYP11A1) [4, 5]. Subsequently, this progestogen is biotransformed either to 17*α*-hydroxypregnenolone or to progesterone via P450 17*α*-hydroxylase (P450c17/CYP17A1) and 3*β*-hydroxysteroid dehydrogenase type 2 (3*β*-HSD2), respectively. Afterward, 17*α*-hydroxypregnenolone is converted to dehydroepiandrosterone (DHEA) by cytochrome P450c17/CYP17A1 [5–7]. The conversion of DHEA to androstenedione via 3*β*-HSD2 or to androstenediol via 17*β*-hydroxysteroid dehydrogenase (17*β*-HSD3) is followed by the biotransformation to T by 17*β*-HSD3 or 3*β*-HSD2, respectively [5]. Furthermore, T is either reduced to 5*α*-dihydrotestosterone (5*α*-DHT) by 5*α*-reductase or to 5*β*-dihydrotestosterone (5*β*-DHT) by 5*β*-reductase [8–10]. Additionally, T can be converted to 17*β*-estradiol (E2) via the aromatase (P450aro/CYP19A1) action, and 17*β*-HSD3 catalyzes the formation of E2 from estrone (Figure 1) [5].

In women, T is produced and secreted by the ovarian stroma, particularly by theca and granulosa cells (25%), the adrenal zona fasciculata (25%), and from circulating androstenedione (50%) [11, 12]. Peripheral tissues such as placenta, liver, skin, prostate, and adipose tissue possess the specific enzymes (or the isoforms) required for the novo synthesis of androgens or their activation from circulating precursors [13]. Furthermore, in the vascular smooth muscle (VSM), airway smooth muscle (ASM), and heart (the tissues that this review is focused on), the expression of some steroidogenic enzymes has been demonstrated. For instance, CYP11A1 and 3*β*-HSD are expressed in cardiac [14, 15], vascular [15, 16], and lung tissue [17]. Nevertheless, CYP17A1, which is required for the conversion of pregnenolone into 17-hydroxypregnenolone, was not found in the heart [14, 15], and it has not been reported in vascular or ASM. Therefore, de novo androgen biosynthesis is unlikely to occur in those tissues. However, the expression of 17*β*-HSD5 in the fetal lung [18, 19] and 17*β*-HSD1,2 in the heart [20] can lead to the biotransformation of pre-existing precursors to T. Interestingly, no significant expression of 17*β*-HSD3 was found in the heart and the lung since this enzyme is considered to be testis-specific [21]. Furthermore, the presence of 5*α*-reductase in the cardiac tissue allows the formation of 5*α*-DHT [20]. Additionally, P450aro has been found in vascular tissues [22, 23], heart [20], and lung epithelial cells [24].

Men usually have much higher levels of T serum concentrations than women. In men from 13 to 80 years old, values of serum T are between 6 and 50 nM [25–27]. 5*α*-DHT (a more potent androgen) represents about 9-10% of the plasma T levels in males of most species [26, 28]. In women, stable serum values of T (0.7–2.5 nM) are maintained except during pregnancy when T concentrations increment (3.5–5 nM) [27]. Also, 5*α*-DHT is essentially produced in peripheral tissues and circulates in very low concentrations in women plasma (0.069 nM) [29].

### 1.2. Nongenomic and Genomic Actions of Androgens

The androgen signaling comprises nongenomic and genomic pathways. The nongenomic effects of androgens are independent of the binding to the cytosolic AR and occur in seconds to minutes [30]. Importantly, these effects are not altered by inhibitors of transcription and seem to be carried out by the androgen binding to plasma membrane lipids or ionic channels [2, 31–35]. Recently, two distinct membrane proteins have been suggested as membrane androgen receptors (mARs): G protein-coupled receptor family C group 6-member A (GPRC6A) and zinc-regulated transporter [Zrt]-protein 9 (ZIP9); both of them may stimulate intracellular pathways via G proteins or mitogen-activated protein kinases (MAPKs) [31, 36–38].

GPRC6A is a member of the C family of G protein-coupled receptors (GPCRs) activated by several ligands such as extracellular Ca^2+^, cations, basic amino acids, osteocalcin, and T [31, 39–41]. Pi et al. in 2010 showed that the stimulation of GPRC6A triggers the inhibitory G protein *α*-subunit (G*α*i), phosphatidylinositol 3-kinase (PI3K), protein kinase C (PKC), proto-oncogene c-Src kinase (Src), and Ras/Raf/mitogen-activated protein kinase kinase (MEK)/extracellular signal-regulated kinase (ERK) signaling pathways [42]. Most recently, the same authors reported that the activation of GPRC6A by testosterone induces cell proliferation and inhibits autophagy through the mammalian target of the rapamycin complex 1 (mTORC1) signaling cascade in prostate cancer cells [43]. ZIP9 is a protein that possesses seven membrane-spanning domains and was first identified as a member of the SLC39A zinc transporter family in Atlantic croaker ovaries [44]. The stimulation of ZIP9 leads to the activation of the Gq protein *α*-subunit (Gq11) in spermatogenic cells, the stimulatory G protein *α*-subunit (G*α*s) in ovarian follicle cells, and the inhibitory G protein *α*-subunit (G*α*i) in prostate cancer cells [36, 37, 44]. Moreover, the activity of ZIP9 (dependent on **T** stimulation) has also been explored in a Sertoli cell line, where this receptor modulates the phosphorylation of ERK1/2 [45]. While the MAPKs signaling pathway can lead to transcription modulation [46], the role of the mARs in the physiology of cardiac and smooth muscle cells is still unrevealed.

The genomic effects of T occur from hours to days and involve the binding of the androgen to a cytosolic androgen receptor (AR). This hormone receptor, also known as NR3C4, is a member of the nuclear receptor family [47, 48]. As in other nuclear receptors, the protein structure of the AR comprises the *N*-terminal domain (NTD), the DNA-binding domain (DBD), the hinge domain (HD), and the ligand-binding domain (LBD) [49]. The stimulation of the AR by T or 5*α*-DHT elicits the dissociation of chaperone proteins and the formation of a complex that is transferred to the nucleus where it modulates gene transcription and protein synthesis [2]. 5*β*-DHT, the other reduced metabolite of T, possesses minor androgenic activity due to a lower binding affinity than 5*α*-DHT [50]. The AR is expressed in several mammalian tissues, including vascular and airway smooth muscles and cardiac myocytes [2, 51–56]. Furthermore, the activity of the AR has been implicated in cardiovascular and respiratory ailments such as vascular ischemia [53], hypertension [57, 58], asthma [52], and cardiac hypertrophy [54].

In the last years, numerous AR splice variants have been molecularly identified and characterized in humans. Although the function of these alternative AR transcripts in the human physiology is not completely understood, these variants have been related to pathological conditions such as prostate cancer (PCa) and androgen insensitivity syndrome (AIS) [59–62]. In 2005, Ahrens-Fath et al. reported the existence of an NTD-truncated AR isoform with a molecular weight of 45 kDa (AR45) in the heart, skeletal muscle, uterus, prostate, breast, and lung [63]. However, the expression level of AR45 compared with the wild-type AR in these tissues is arguable since a semiquantitative RT-PCR was performed by Ahrens-Fath et al. Also, this receptor variant is expressed in the normal prostate tissue and in human prostate adenocarcinoma derived from the left supraclavicular lymph node metastasis (LNCaP) cells [64, 65]. Additionally, it has been shown that AR45 may repress or stimulate wild-type AR activity [63]. Interestingly, 12 AR variants lacking the LBD (ARV1-12) have been identified in PCa cell lines [64–67]. Among all the ARV isoforms, ARV7 (also known as AR3) has gained relevance due to its demonstrated capability of mediating constitutively AR functions, i.e., constitutive gene transcription in the absence of androgen stimuli. Moreover, ARV7 has been suggested as a predictive biomarker in castrate-resistant PCa since it promotes cancer progression and androgen depletion-resistant growth by regulating serine/threonine kinase 1 encoding gene (AKT1) [64–67]. In spite of the emerging evidence about AR splice variants, further studies are imperative in order to elucidate the possible expression and the physiological role of these alternative transcripts in vascular and airway smooth muscles and cardiac muscle.

Noteworthy, it has been proposed that androgen nongenomic and genomic actions may converge. For instance, in the vascular smooth muscle, the regulation of K^+^ channels is dependent on nongenomic and genomic effects of androgens [30]; however, cellular mechanisms and signaling pathways displayed in both types of actions are entirely different and carried out by distinct effector proteins.

### 1.3. Androgens and Vascular, Airway, and Cardiac Muscles

Vascular smooth muscle (VSM), airway smooth muscle (ASM), and cardiac muscle (CM) cells are excitable entities, with the primary function of contracting and relaxing [68]. Several research groups have shown that androgens interact with the contraction and relaxation mechanisms of different muscular cell types from distinct species through nongenomic and genomic effects.

With respect to nongenomic actions, in the VSM, numerous authors have reported that androgens induced vasorelaxation in different arteries [69–74]. In this regard, in the ASM, our group and others have observed that DHEA, T, 5*α*-DHT, and 5*β*-DHT induced-relaxation through nongenomic actions [33, 75–78].

In relation to the genomic actions, it has been reported that T  and DHT  induced in the VSM, the genic expression of proteins such as adenylyl cyclase (AC), Ca^2+^-activated K^+^ channels of high conductance (BK_Ca_), and L-type voltage-dependent Ca^2+^ channels (L-VDCCs) [73, 79]. Most recently, we found in the ASM that T augmented the expression of *β*_2_-ARs, favoring an increase in the relaxing response to salbutamol [51]. In the CM, it has been described that androgens (via a genomic effect) increased the expression of the voltage-dependent delayed rectifier K^+^ channel 1.5 (K_V_1.5), leading to shortening of the action potential duration in mice ventricular cardiomyocytes [80], and also enhanced the expression of K_V_1.7 diminishing the QT intervals in rats [81]. Testosterone nongenomic and genomic actions and their association with the adrenergic system of vascular, airway, and cardiac myocytes are discussed in the next sections of this manuscript.

### 1.4. Adrenergic Receptors in Vascular, Airway, and Cardiac Muscles

Under physiological conditions, the adrenergic system plays a critical role in regulating vascular, airway, and cardiac function. In the VSM and CM, sympathetic innervation modulates contraction [82] and the intrinsic conduction system [83, 84], respectively. The ASM tone is partly regulated through circulating catecholamines such as epinephrine released from the adrenal medulla [85, 86]; this hormone acts as an adrenergic receptor agonist. The adrenergic receptors or adrenoceptors are members of the superfamily of G protein-coupled receptors (GPCRs) and modulate several pathways through effectors such as AC or phospholipase C (PLC) [87]. Adrenergic receptors have been classified into three major categories: alpha-1-adrenergic receptors (*α*_1_-ARs), alpha-2-adrenergic receptors (*α*_2_-ARs), and beta-adrenergic receptors (*β*-ARs). Moreover, each of these groups has been further subclassified into multiple subtypes defined by the differences in their genetic sequences and their pharmacological action: *α*_1A_, *α*_1B_, *α*_1D_, *α*_2A_, *α*_2B_, *α*_2C_, *β*_1_, *β*_2_, and *β*_3_ [88, 89].

*α*_1_-ARs are coupled to a heterotrimeric Gq protein and PLC signaling pathway. PLC triggers the formation of inositol 1,4,5-trisphosphate (IP_3_) and diacylglycerol (DAG), resulting in the increase of the intracellular Ca^2+^ concentration ([Ca^2+^]_i_) and the activation of protein kinase C (PKC) [87, 90–92]. Also, the stimulation of *α*_1_-ARs promotes an extracellular Ca^2+^ influx through voltage-dependent Ca^2+^ channels (VDCCs) [93] and triggers extracellular signal-regulated kinases 1 and 2 (ERK1/2) [94, 95]. In humans, *α*_1A_, *α*_1B_, and *α*_1D_ adrenergic receptors are encoded by distinct genes located on chromosomes 8, 5, and 10, respectively [87]. The three subtypes of *α*_1_-ARs are present in most blood vessels modulating smooth muscle contraction and vascular tone. *α*_1A_ is the most prevalent subtype in human arteries; nevertheless, the expression levels of *α*_1_-ARs depend on the vascular bed studied. *α*_1D_-AR subtype predominates in large conduction vessels as the aorta and carotid arteries, whereas *α*_1A_-AR subtype is involved in regulating vascular tone of mesenteric, splenic, pulmonary, and caudal (in mice and rats) arteries controlling organ blood flow [96–101]. While *α*_1A_ and *α*_1D_-ARs are the main subtypes involved in vascular contractions, *α*_1B_-AR subtype is also expressed in several blood vessels, and it was thought that it did not require extracellular Ca^2+^ to activate smooth muscle contraction [99, 100]. Unfortunately, studies related to *α*_1B_-AR function in the VSM have been restrained by the lack of selective antagonists. However, this receptor subtype has been proposed to be involved in the regulation of systemic BP [102–104] and coronary blood flow [105].

The evidence of *α*-ARs in the ASM is also present; nonetheless, these receptors seem not to be relevant in the functionality of this tissue. In this context, norepinephrine-induced contraction has been observed in guinea pig [106, 107], rabbit, cat, and rat [107] tracheal preparations but only after *β*-AR blockade. Interestingly, Kneussl and Richardson in 1978 found that human and dog ASM did not contract in response to norepinephrine, unless they were previously stimulated with histamine or KCl [108]. These insights confirm the predominance of relaxant *β*-AR function in the ASM of most mammals. However, in 1985, Montaño et al. revealed that the *Erythrocebus patas* monkey possesses *α*-AR predominance in this tissue [109].

In cardiomyocytes of species such as mice, rats, and humans, all three *α*_1_-AR mRNAs have been detected with the predominance of *α*_1A_- and *α*_1B_-AR subtypes [105, 110–112]. Although the stimulation of these receptors in cardiomyocytes can evoke muscle contraction, most works have been focused only on ventricular heart sections. In this context, it has been observed that norepinephrine and epinephrine can induce positive inotropic and chronotropic effects in the right atrium from mice, probably through *α*_1_-AR signaling [113]. Nevertheless, different studies have shown that, in the heart, *α*_1_-ARs are mainly involved in processes such as hypertrophic responses, upregulation of myosin light chain-2, modulation of the atrial natriuretic factor (ANF), and heart failure [114–120].

*α*_2A_, *α*_2B_, and *α*_2C_ adrenoceptor genes are located on human chromosomes 10, 4, and 2, respectively. The encoded products share about 50% of amino acid identity and show the same affinity for norepinephrine and epinephrine [121]. All *α*_2_-ARs are coupled to the pertussis toxin-sensitive G proteins such as Gi/Go and to the inhibition of the AC. The consequence of the inhibition of this enzyme is a decrease in the production 3′, 5′-cyclic adenosine monophosphate (cAMP), reducing the activity of protein kinase A (PKA) [121–125]. Additionally, Gi/Go signaling cascade modulates Ca^2+^ [126, 127] and K^+^ [128, 129] channels without the involvement of other second messengers. VSM cells express all subtypes of *α*_2_-ARs, and their stimulation is related to contraction and vasopressor effects [124, 130–132]. *α*_2A_-Adrenoceptor is the most predominant subtype in this tissue and participates in the regulation of the muscular tone in the aorta [133] and carotid (possibly controlling cerebral blood flow) [134, 135] and mesenteric arteries [136] and in the peripheral vasoconstriction related to the skin blood flow [137]. *α*_2B_-AR is more involved in the vascular tone of smaller arteries [138] but contributes to BP regulation to a greater extent than *α*_2A_- and *α*_2C_-ARs [139]. Furthermore, *α*_2_-adrenoceptors seem to play a minor role in cardiac contractility compared to *β*- and *α*_1_-ARs. Recently, it was demonstrated in ventricular cardiomyocytes that the stimulation of *α*_2_-ARs could modify [Ca^2+^]_i_ and induce myocardial contraction [140, 141].

*β*-ARs, like all other adrenergic receptor subtypes, are composed of seven transmembrane spanning helices. The three subtypes (*β*_1_, *β*_2_, and *β*_3_) are found in VSM [87], ASM [142], and CM [143] cells. Their coding genes are located in human chromosomes 10, 5, and 8, respectively [87, 144, 145]. The stimulation of the *β*-AR mediates the activation of AC and the subsequent increment in the production of cAMP [146, 147]. In its active state, *β*-AR is associated with the *α*-subunit of Gs protein. In the VSM, the activation of *β*-ARs induces the relaxation of the tissue, regulating the peripheral vascular resistance and controlling the organ blood flow and vascular tone [87]. Among all *β*-ARs, *β*_2_-AR is the predominant expressed subtype in most vascular beds, while a minor proportion of *β*_1_-ARs is also present. Apparently, *β*_1_-adrenoceptors play an essential role in the function of coronary and cerebral arteries [87, 148–151]. *β*-Adrenoceptors also occur in endothelial cells where they mediate vasodilation through nitric oxide (NO) production [152]. In airways, *β*_2_-agonists are well known as the most effective bronchodilators. The *β*_2_-agonist binding to the *β*_2_-AR in the cell membrane of the ASM triggers the formation of cAMP by the action of the AC [153–155]. Subsequently, the increment of the cAMP levels activates PKA, a phosphorylating protein, which favors K^+^ channel opening and bronchorelaxation [156]. In the heart, *β*_1_ and *β*_2_ are the most valuable adrenoceptor subtypes with a predominance of *β*_1_-ARs over *β*_2_-ARs (ratio of ∼80/20). The stimulation of these receptors in cardiomyocytes mediates positive chronotropic, inotropic, and lusitropic effects [157–159]. Gs-PKA signaling in cardiomyocytes promotes the phosphorylation of phospholamban (PLB), L-VDCCs, ryanodine receptors (RyRs), and cardiac myosin-binding protein C leading to an increase in [Ca^2+^]_i_ and favoring muscle contraction [160]. Interestingly, it has been demonstrated that a sustained activation of *β*_1_-ARs may induce cardiotoxic effects, and *β*_2_-ARs switch their natural Gs coupling to Gi protein coupling, opposing the positive *β*_1_-AR effects [157, 161, 162].

It is well known that adrenoceptors play a key role in maintaining vascular, airway, and cardiac muscular function. In this regard, the modulation by T of the adrenergic receptor signaling pathway has been investigated, and the observed effects appear to be dependent on the studied tissue and the predominance of the adrenergic receptor subtypes either favoring muscle relaxation or contraction [51, 79, 163, 164]. This review focuses on the effects of T on the adrenergic system in the vascular, airway, and cardiac muscles and its relevance in pathological processes related to this system.

## 2. Vascular Smooth Muscle

The maintenance of the vascular tone is due to the balance between vasoconstriction and vasorelaxation modulated by several neurotransmitters and hormones [165]. The VSM found in the medial layer of the blood vessels is responsible for controlling vascular tone and blood pressure (BP) [166]. The regulation of the VSM membrane potential and the vascular tone is mainly determined by Ca^2+^ and K^+^ channels [167, 168]. The main K^+^ channels expressed in the VSM are the voltage-dependent delayed rectifier K^+^ channels (K_V_), Ca^2+^-activated K^+^ channels of high conductance (BK_Ca_), ATP-sensitive K^+^ channels (K_ATP_), and inward-rectifier K^+^ channels (K_IR_) [169, 170]. VSM constriction is caused by increments in [Ca^2+^]_i_ [171]. Vasoconstrictor agonists act on GPCRs coupled to the _q_*α* subunit (GPCR-_q_*α*) such as *α*_1A_-, *α*_1B_-, and *α*_1D_-ARs, bradykinin, histamine H_1_, and thromboxane-A_2_ receptors, among others [172–174]. These receptors activate the PLC enzyme and IP_3_ signaling pathway, inducing the release of Ca^2+^ from the sarcoplasmic reticulum (SR) and the influx of this ion through VDCCs [175]. In the VSM, two major subtypes of VDCCs with distinct electrophysiological properties are present. L-VDCCs are activated by large depolarizations and inactivated relatively slowly. T-type voltage-dependent Ca^2+^ channels (T-VDCCs) are activated by small depolarizations and inactivated rapidly [176, 177].

**Figure 2:**
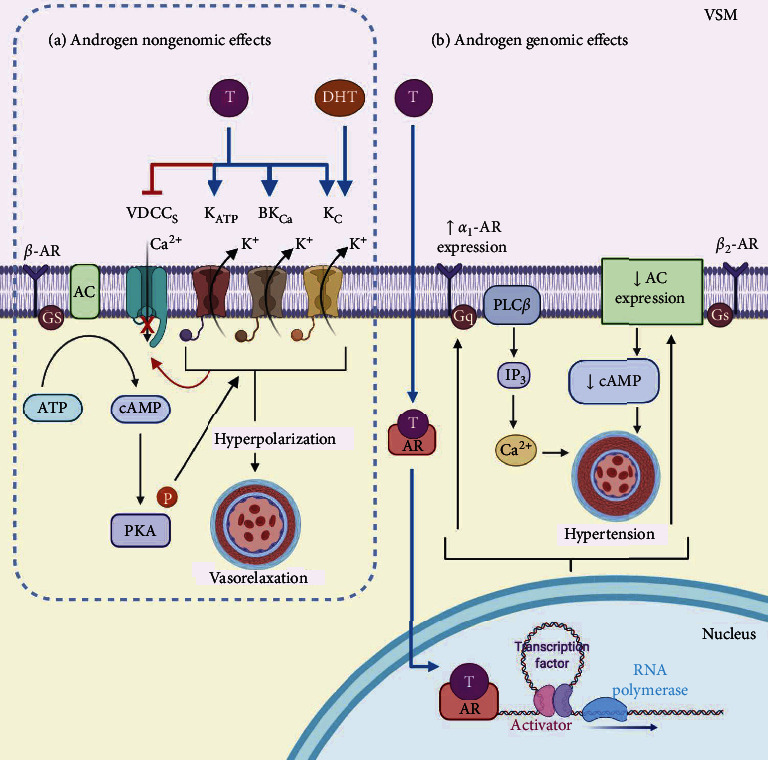
Androgen effects on the adrenergic system in the vascular smooth muscle (VSM). (a) Stimulation of the *β*-adrenergic receptor (*β*-AR) leads to an increase in the activity of the K^+^ channels and to plasma membrane hyperpolarization. *β*-AR receptor is coupled to a Gs protein (Gs) that activates adenylyl cyclase (AC), which enhances the synthesis of 3′,5′-cyclic adenosine monophosphate (cAMP) and consequently promotes the protein kinase A- (PKA-) induced phosphorylation of the K^+^ channels. K^+^ channel phosphorylation increases their open probability and evokes membrane hyperpolarization that closes Ca^2+^ channels, leading to vasorelaxation. Testosterone, via a rapid response (nongenomic), activates ATP-sensitive K^+^ channels (K_ATP_), Ca^2+^-activated K^+^ channels of high conductance (BK_Ca_), and voltage-dependent delayed rectifier K^+^ channels (K_V_). Dihydrotestosterone (DHT, a reduced metabolite of T) enhances the activity of the K_V_ channel. T also blocks VDCCs. Androgen-induced vasorelaxation mediated by the activation of K^+^ channels and the blockade of VDCCs might improve the response of *β*-AR signaling. (b) The genomic androgen receptor (AR) signaling involves androgen crossing the plasma membrane, entering the cytoplasm, dissociation of chaperone proteins, and binding to its cytosolic receptor. AR stimulation by T  results in a decrement of AC expression and a reduction of cAMP synthesis. Moreover, T increases the *α*_1_-adrenergic receptor (*α*_1_-AR) expression. This receptor is coupled to a Gq protein (Gq), which, through phospholipase C_*β*_ (PLC_*β*_), catalyzes the formation of inositol-1, 4, 5-triphosphate (IP_3_) and triggers intracellular calcium release from the sarcoplasmic reticulum (SR). The genomic effects of T favor vasoconstriction in the VSM and might lead to hypertension development.

Moreover, VDCCs are not the only source of extracellular Ca^2+^. The influx of this ion is also carried out by nonselective cation channels such as receptor-operated Ca^2+^ channels (ROCCs), store-operated Ca^2+^ channels (SOCCs), and transient receptor potential (TRP) channels. The Ca^2+^ influx exerted by these channels is thought to be triggered by agonists such as norepinephrine, vasopressin, and acetylcholine via GPCRs linked to the phospholipase C_*β*_ (PLC_*β*_) signaling pathway and the formation of IP_3_ and DAG. This last second messenger regulates the activity of ROCCs, and IP_3_ induces depletion of internal Ca^2+^ stores leading to capacitative Ca^2+^ entry through SOCCs [178–181]. Additionally, TRP channels have been classified as ROCC subtypes, and transient receptor potential canonical channels 3, 6, and 7 (TRPC3, TRPC6, and TRPC7) have been shown to be susceptible to DAG stimulation promoting its opening and contributing to Ca^2+^ influx. Afterward, Ca^2+^ complexes with calmodulin to activate myosin light-chain kinase (MLCK) causing vasoconstriction [168, 182]. Conversely, the decrease in cytosolic Ca^2+^ leads to vasorelaxation [183]. Vasodilator agonists that stimulate GPCRs coupled to the _s_*α* subunit (GPCR-_s_*α*) such as *β*_1_- and *β*_2_-ARs, histamine H_2_, prostaglandin E_2_, and adenosine A_2_ receptors, among others [184], induce the synthesis of cAMP and 3′, 5′-cyclic guanosine monophosphate (cGMP); therefore, they activate PKA and protein kinase G (PKG), respectively [185], leading to a decrease in the vascular tone [183]. In the last two decades, the evidence about the relationship between androgens and vascular reactivity has increased. The nongenomic effects of T in the VSM can be due to its action on ion channels resulting in vasorelaxation. In 1996, Perusquía et al. postulated that T, 5*β*-DHT, and 5*α*-DHT induced vasorelaxation in the rat aorta [186]. Later on, the same group observed that T was capable of blocking the extracellular Ca^2+^ influx inducing vasorelaxation of the precontracted human umbilical artery [72]. More recently, it was demonstrated that 5*β*-DHT and T induced vasorelaxation by blocking L-VDCCs in the rat thoracic aorta [70]. In addition to blocking Ca^2+^ entry through L-type Ca^2+^ channels, T  is capable of activating K^+^ channels. The efflux of K^+^ evokes membrane hyperpolarization and closes Ca^2+^ channels leading to vasorelaxation in pig [187] and rabbit [188] coronary arteries. In this regard, different types of K^+^ channels have been proposed as targets for T  modulation. In the dog coronary artery [189] and rat aorta [190], K_ATP_ channels have been shown to be involved in the T-associated relaxant effect. BK_Ca_ channel activation in the human internal mammary artery [191] and pig coronary artery [187] is also implicated in T-induced vasorelaxation. Moreover, Saldanha et al. demonstrated that T produced relaxant responses in human umbilical artery rings precontracted with serotonin (5-HT), histamine, and KCl, and these effects were dependent on both BK_Ca_ and K_V_ channel activity (Figure 2(a)). They also studied the long-term effects of androgens in the same model, founding that DHT, through genomic actions, decreased the mRNA expression of the *α*-subunit of L-VDCC and upregulated the *β*_1_-subunit of BK_Ca_, favoring relaxation [73].

### 2.1. The Effects of Testosterone on Adrenergic Receptors in the Vascular Smooth Muscle

Sex differences in cardiovascular diseases, i.e., hypertension, have been broadly studied. Men are more likely to develop hypertension or coronary heart disease (CHD) than women [192–194]. Hypertension is defined as persistent systolic BP ≥ 140 mmHg and or diastolic BP ≥ 90 mmHg, according to 2018 ESC/ESH guidelines [195]. The World Health Organization has rated hypertension as one of the deadliest causes of premature death worldwide due to its asymptomatic behavior that can result in concomitant diseases after years. In this regard, sex differences in the development of hypertension have been reported. Female sex hormones, such as estrogens, have been widely implicated in the hypertension-related gender differences [57]; however, several authors have pointed out a prohypertensive role for androgens [58]. Studies in humans and castrated rats revealed that androgens exert a prohypertensive effect, while estrogens appear to oppose the increase in BP [196]. In this context, Torres et al. found in castrated male Wistar rats an increment in aortic vasodilation, indicating a sex hormone influence [197]. Another research group observed that gonadectomized hypertensive rats, both males and females, showed a reduced BP, and the administration of T restored it in the castrated male experimental group [198]. Moreover, it has been proposed that the effect of T on VSM does not benefit a state of relaxation but rather favors vasoconstriction. Fluctuations in androgen concentrations throughout life stages can affect the vascular tone, and T may contribute to developing hypertension [58]. In this sense, hyperandrogenism (HA) in pre- and postmenopausal women has been associated with an unfavorable metabolic profile, obesity, and hypertension [199–201]. HA is defined as an excess of androgen production and secretion by adrenal glands or the ovaries [202]. Moreover, the development of HA in females has shown to be associated with ovarian disorders, e.g., ovarian hyperthecosis (OH) [203], virilizing ovarian tumors (VOTs) [204], and polycystic ovary syndrome (PCOS) [199–201]. PCOS is one of the most common endocrine disorders affecting women of reproductive age [205]. The metabolic phenotype in PCOS is characterized by increased LH compared with the follicle-stimulating hormone (FSH) and HA [205, 206]. Furthermore, evidence points out that hyperandrogenemia in women suffering from PCOS is associated with an increased systolic and diastolic BP, and this relation is independent of other risk factors such as obesity and insulin resistance [207].

During aging, the vascular tone is led to vasoconstriction, and *β*-ARs have been proposed as targets of several drugs related to hypertension disease [208]. Aged animals have a weak vascular response to *β*-AR agonists, and possibly, mechanisms of the *β*-AR signaling pathway are altered [209]. Vascular tone is modulated through the action of the sympathetic nervous system (SNS) on *β*-ARs promoting the increase in cAMP levels [142, 210]. It has been reported that androgens promote vasoconstriction by increasing catecholamine (mainly norepinephrine) levels [57]. In 2005, Martin et al. demonstrated that the adrenergic system (through norepinephrine action) reduced the mean arterial pressure in castrated male spontaneously hypertensive rats (SHR) [211]. In other studies, vascular tone at different stages of rat growth was compared to explore the role of T in *β*-adrenergic-induced vasodilation [79]. In aortic rings of mature rats, vasorelaxation response induced by isoproterenol (a well-known unspecific *β*-adrenergic agonist) showed an impairment of this response compared to aortic rings obtained from younger rats. According to the authors, this impaired relaxing response could be related to higher plasma T-levels in older rats. The authors elegantly demonstrated that T reduced the *β*-AR-elicited vasorelaxation without any alteration in the expression of the *β*_2_-AR but interfering downstream in the signaling cascade. Furthermore, the authors exhibited that T (via a genomic effect) diminished the expression of AC and yielding of cAMP in castrated rats [79]. These findings point out that changes in the levels of T could lead to high BP and hypertension.

Furthermore, the vessel tone is also regulated by *α*-ARs. These receptors promote vasoconstriction and might contribute to hypertension development [92]. In this context, the modulation of the *α*_1_-AR by T has been reported. Testosterone replacement therapy increased BP in gonadectomized SHR and the number of *α*_1_-ARs in the tail artery [164]. Furthermore, in 1999, it was found that the incubation for 24 hr with T (0.1 nM–1 *μ*M) increased the abundance of *α*_1B_-AR mRNA in VSM cells through a genomic action. The same study reported that glucocorticoids, such as dexamethasone, increased catecholamine-mediated vasoconstriction due to an increased *α*_1B_-AR expression [212]. In this context, T is not the only steroid hormone related to vascular physiology. High concentrations of glucocorticoids (such as cortisol) promote the retention of sodium and decrease the activity of prostaglandins leading to a contracted state of the VSM [213].

Although the regulation through norepinephrine of the vessel tone is essential for both females and males, the existence of sex differences in vessel vasoconstriction and vasodilatation has been reported. In 2017, Al-Gburi et al. demonstrated that the *α*-adrenergic vasoconstriction was weaker in female than male rats. They also found that the stimulation of *β*_1_-, *β*_2_-, and *β*_3_-ARs evoked a greater response of relaxation in females than in males [214]. The diminished vasoconstriction and the enhanced vasorelaxation were due to the upregulated expression of the *β*_1_- and *β*_3_-ARs mainly in an endothelial location in female rats. Later on, Riedel et al. confirmed the overexpression of the *β*_1_- and *β*_3_-ARs in endothelial cells of the blood vessel by the action of the estrogens. The endothelial adrenergic stimulation caused an enhanced NO-dependent vasorelaxation in female rats [215], counteracting the vasoconstrictive outcomes modulated by the *α*-ARs [215]. These findings could explain very well the sex and age differences on the role of the adrenergic response in the VSM.

In conclusion, **T** reduces the *β*-AR-elicited vasorelaxation by interfering downstream in the signaling pathway and upregulates the *α*-AR expression (Figure 2(b)). These hormonal effects are carried out principally through genomic actions leading to vasoconstriction and might be involved in the development of hypertension. Nevertheless, androgen nongenomic actions have opposite outcomes in the VSM, yielding their effects to vasorelaxation. However, the genomic actions of androgens (long-term effects) seem to be the predominate deleterious effects favoring hypertension. Therefore, the possible use of androgens, due to their nongenomic actions, as a therapeutic tool for the treatment of hypertension could not be appropriated based on their long-term genomic actions.

## 3. Airway Smooth Muscle

The maintenance of proper air flux through the airways results from the balance between contraction and relaxation of the ASM. The response of the ASM to physiological and pathophysiological stimuli determines the airway caliber in order to regulate the airflow [216]. The basal tone of the ASM is maintained by the influx and efflux of Ca^2+^ across the cell membrane, keeping an intracellular basal Ca^2+^ concentration (_b_[Ca^2+^]_i_) around 100–150 nM [33, 77, 217–219]. The SR, ion channels, GPCRs, ATPases, and other mechanisms preserve _b_[Ca^2+^]_i_ in the ASM cells. The mechanisms responsible for the Ca^2+^ influx are carried out by transient receptor potential canonical 3 (TRPC3), L-VDCCs and T-VDCCs, ROCCs, SOCCs, and reverse-mode Na^+^/Ca^2+^ exchanger (NCX_REV_) [77, 219]. Endogenous agonists such as acetylcholine, histamine, and leukotrienes act through the GPCRs-_q_*α* pathway. These receptors activate the PLC_*β*_ enzyme, which catalyzes the formation of DAG and IP_3_, favoring SR Ca^2+^ release through the IP_3_ receptor [220]. Increased Ca^2+^ in the cytosol promotes the release of more Ca^2+^ (Ca^2+^ sparks) through RyRs; this event is known as Ca^2+^-induced Ca^2+^ release (CIRC) [221, 222]. Increase in [Ca^2+^]_i_ is restored by two ATPases: sarcoplasmic reticulum Ca^2+^-ATPase (SERCA) and plasma membrane Ca^2+^-ATPase (PMCA) [223, 224]. Airway smooth muscle relaxation is predominantly mediated by the sympathetic system. Circulating epinephrine is more important in mediating relaxation in human airways than norepinephrine. In the ASM, *β*_2_-AR is the main adrenoceptor subtype responsible for the bronchodilator effect [156]. Activation of this receptor triggers the formation of cAMP and, consequently, the activation of PKA [156]. PKA-mediated phosphorylation modulates proteins involved in the control of the airway muscle tone by regulating the Ca^2+^ availability and inactivating myosin light-chain kinase [225]. Furthermore, it is well known that the activation of the *β*_2_-AR favors hyperpolarization and relaxation of the ASM through the opening of different K^+^ channels [156, 226]. In the ASM, the main K^+^ channels are the Ca^2+^-activated K^+^ channels (K_Ca_) and K_V_ [227, 228]. K_Ca_ are activated by increases in [Ca^2+^]_i_ and through the cAMP-PKA signaling pathway [229, 230]. There are three subfamilies of K_Ca_, all of them occurring in airways: high conductance (BK_Ca_), intermediate conductance (IK_Ca_), and low conductance (SK_Ca_) [229]. Moreover, K_V_ have been characterized as K_V_1.2, K_V_1.5, and K_V_7.5 in the ASM [227, 231]. Several agonists can lead to the bronchodilation of the ASM involving the opening of distinct K^+^ channels. In this regard, it has been shown that the most critical channels in bronchodilation induced by 5-HT and ATP are BK_Ca_ [226, 228]. Most recently, our research group demonstrated that both K_Ca_ and K_V_ are implicated in salbutamol-induced relaxation in guinea pig airways [51].

Sex hormones play a role in the development of lung diseases. Androgens have been associated with asthma. During puberty, girls are more vulnerable to present asthma symptoms than boys, until the fifth life decade, when men become more susceptible than women [232, 233]. It has been reported that the variations in sex hormones during the menstrual cycle, hormone replacement therapy, and pregnancy have an influence in asthma patients [234–236]. Asthma is a chronic and inflammatory disease, characterized by hyperresponsiveness of the airways (AHR). This phenomenon is presented as an increased reactivity of the ASM to different agonists that leads to exaggerated bronchoconstriction. In addition, this disease is conducted by a type 2 immune response through eosinophils, basophils, mast cells, etc. [237]. However, not all asthma patients course with type 2 inflammation; instead, they can display interleukin-17- (IL-17-) mediated neutrophil inflammation [238].

Several studies have exposed that T induces a potential ASM relaxation effect through a nongenomic effect. An early work was conducted in the rabbit tracheal smooth muscle previously contracted with cholinergic agonists. The addition of T relaxed the ASM in an epithelium-dependent way involving NO production [78]. Later on, it was found that T relaxed precontracted guinea pig and bovine tracheal smooth muscles in an epithelium-independent way by blocking L-VDCCs [76]. In this context, our group demonstrated that T blocked L-VDCCs and SOCCs in the guinea pig ASM [34]. Additionally, the same study revealed that T induced the synthesis of prostaglandin E_2_ (PGE_2_), the main relaxing prostanoid in the airways [34]. Our studies pointed out that the blockade of the L-VDCCs and SOCCs and the production of PGE_2_ are the main components of the T-induced relaxation in guinea pig precontracted airways. Then, we observed that T did not only relax the guinea pig ASM but lowered _b_[Ca^2+^]_i_ and the muscular tone through the inhibition of L-VDCCs and TRPC3 [77, 219]. Most recently, our research group found that T interfered with the IP_3_ receptor, decreasing the cholinergic-induced guinea pig ASM contraction [33]. Noteworthy, all the previously mentioned effects of T on ASM were carried out through nongenomic effects. Likewise, T, via a genomic action, negatively regulates type 2 inflammation and the expression of IL-17A [239, 240]. Furthermore, it was found that androgens, via AR activation, mediate the regulation of intracellular Ca^2+^ increment induced by proinflammatory cytokines such as tumor necrosis factor alpha (TNF-*α*) or interleukin-13 (IL-13) in the human ASM [52]. All these androgen effects contribute to diminishing the ASM reactivity and favor the absence of asthma symptoms.

### 3.1. The Effects of Testosterone on Adrenergic Receptors in the Airway Smooth Muscle

**Figure 3:**
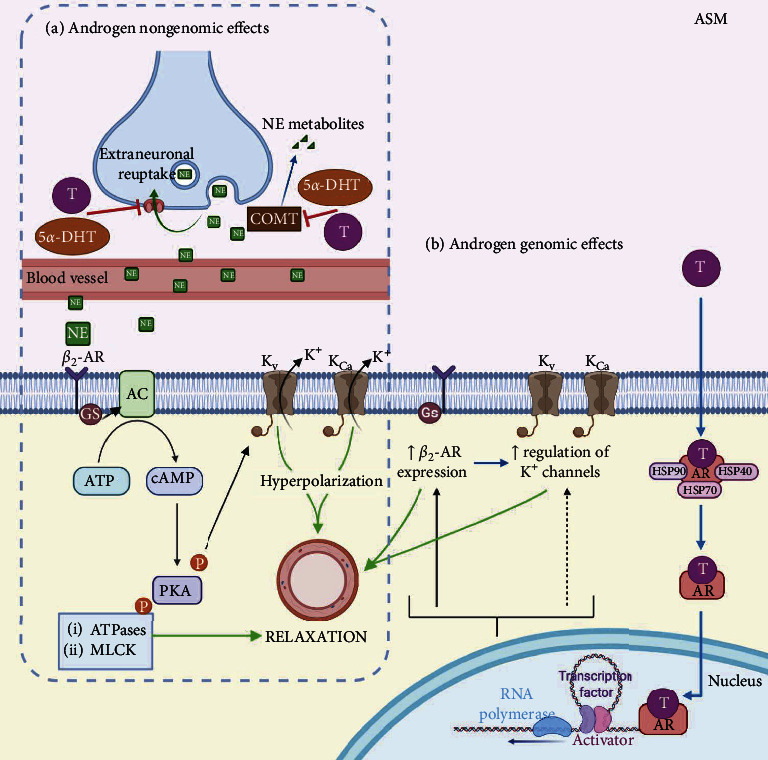
Androgen effects on the adrenergic system in the airway smooth muscle (ASM). (a) Testosterone (T), through a nongenomic effect, potentiates the relaxation induced by the *β*_2_-adrenergic agonist through the inhibition of catechol-O-methyl transferase (COMT) or by the abolition of extraneuronal uptake of catecholamines. The inhibition of these mechanisms by T or 5*α*-dihydrotestosterone (5*α*-DHT, a reduced metabolite of T) leads to the accumulation of catecholamines such as norepinephrine (NE). The *β*_2_-adrenergic agonist activates adenylyl cyclase (AC), leading to the activation of protein kinase A (PKA), which phosphorylates K^+^ channels, ATPases, and myosin light-chain kinase (MLCK). These targets promote the relaxation of the ASM. (b) The androgen receptor (AR) signaling involves androgen crossing the plasma membrane, entering the cytoplasm, dissociation of chaperone proteins, and binding to the AR. Testosterone stimulation increases the *β*_2_-adrenergic receptor (*β*_2_-AR) expression in the guinea pig ASM and upregulates the Ca^2+^-activated K^+^ channels (K_Ca_) and the voltage-dependent delayed rectifier K^+^ channels (K_V_). K_Ca_ are activated by increases in intracellular Ca^2+^ and through the cAMP-PKA signaling pathway. In the ASM, the main K^+^ channels are the Ca^2+^-activated K^+^ channels of high conductance (BK_Ca_). The K_V_ channel subtypes characterized in the ASM are K_V_1.2 and K_V_1.5. T (nongenomic and genomic effects) favors the relaxation of the ASM and might contribute to decreasing asthma symptoms.

Treatment with *β*-agonists to reverse airway obstruction, as seen in asthma and chronic obstructive pulmonary disease (COPD), has an essential role in controlling exacerbations. Therapeutically, there are two types of *β*-agonists: long-acting *β*-agonists to manage asthma together with glucocorticoids and short-acting *β*-agonists to relieve exacerbations [241]. Physiologically, the circulating catecholamines mediate the relaxation of the airways in humans. The androgen effects on the expression or function of the *β*-AR in the ASM have been scantly studied. In 1972, Salt and Iverson reported that T, via a nongenomic action, acted as an inhibitor of the extraneuronal uptake for catecholamines in the CM [242]. In this context, it was found that T potentiated the relaxation induced by isoprenaline (a nonselective *β*-adrenergic agonist) in pig bronchus, also via a nongenomic effect. The authors claimed that the potentiation effect observed was due to the inhibition of catechol-O-methyl transferase (COMT) or abolition of extraneuronal uptake [243]. In 2008, Bordallo et al. showed that 5*α*-DHT (a reduced metabolite of T) potentiated the relaxation induced by salbutamol, a *β*_2_-adrenergic agonist, in the bovine tracheal ASM [76]. However, the effect of 5*α*-DHT seemed not to be related to a direct interaction with *β*_2_-AR. Although the authors did not define the cause of the potentiation, it might be related to the inhibition of both the uptake of catecholamines and COMT (Figure 3(a)). Most recently, our group studied the genomic effects of T on *β*_2_-AR. We found that chronic guinea pig ASM exposure to T augmented the expression of *β*_2_-AR and evoked an increase in the relaxing responses to salbutamol (Figure 3(b)). Interestingly, this effect was abolished by flutamide (antagonist of the AR) [51]. We also observed that T potentiated salbutamol-induced potassium currents (I_K_) involving the K_V_ and K_Ca_ upregulation (Figure 3(b)). Contrasting with other studies in the VSM [79], we did not find any modification of the adenylyl cyclase 6 (AC-6, the main isoform in the ASM) expression in tissues chronically exposed to T [51]. In summary, in the ASM, T and its metabolites, through nongenomic and genomic actions, have complementary effects. Consequently, androgens might play an important role as potential physiological modulators of the ASM tone, facilitating relaxation via *β*_2_-AR, and therefore could be a therapeutic alternative for asthma treatment, although further research is needed (Figure 3).

## 4. Cardiac Muscle

Traditionally in the CM, autonomic control is derived by extrinsic signals or electrical stimulation of peripheral nerves. Moreover, neurocardiac control is maintained by an extensive network of intrinsic cardiac neurons, i.e., the intrinsic cardiac nervous system (ICNS) [244–246]. The ICNS comprises collections of neuronal somas residing on supraventricular tissues and the epicardial surface. This system is also composed by connecting nerve fibers known as ganglionic plexuses (GPs) [222, 246]. GPs are distributed in 5–7 regions comprising the right dorsal atrial, ventral right atrial, left dorsal, ventral left atrial, middle dorsal, right coronary, and left coronary plexuses [247]. Neuronal activity is modified by the activation of sensory nerves [248] and neuroactive chemicals, including acetylcholine, histamine, *α*- and *β*-adrenergic agonists, NO, neuropeptide Y (NPY, coreleased alongside norepinephrine), and calcitonin gene-related peptide (CGRP) [246, 249].

Similar to other muscular cells, [Ca^2+^]_i_ determines the contractile function of the heart through distinct Ca^2+^-handling proteins. In the sinoatrial node, the pacemaker cells start depolarization of the cardiac myocytes. This process is regulated by the parasympathetic nervous system (PNS) and the SNS [250]. The self-depolarization produces action potentials along the CM, allowing the influx of Ca^2+^ through L-VDCCs and T-VDCCs. Furthermore, the Ca^2+^ influx elicits calcium release from the SR via RyR isoform 2 (RyR_2_) [251]. Cardiac contraction results from a sudden increase in [Ca^2+^]_i_ and the formation of the Ca^2+^-calmodulin complex with the further activation of MLCK. Afterward, Ca^2+^ is sequestered to the SR by SERCA, and the cell takes it out by the Na^+^-Ca^2+^ exchanger in its forward mode (NCX). In addition, K_Ca_ channels are activated, leading to membrane hyperpolarization. These are the main mechanisms responsible for CM relaxation [252, 253]. Physiologically, catecholamines, through *β*-ARs, induce the synthesis of cAMP and the activation of PKA. This kinase promotes cardiac contraction by phosphorylating the L-VDCCs and RyR_2_ since they increase their open probability and therefore the increment in [Ca^2+^]_i_ [254, 255]. PKA is also capable of evoking the relaxation of the CM by phosphorylating PLB, allowing SERCA to pump Ca^2+^ into the SR more rapidly [256]. In human ventricular cardiomyocytes, *β*_1_- and *β*_2_-ARs enhance cardiac frequency and contractility; meanwhile, *β*_3_-ARs mediate negative inotropic effects [257]. The *β*_2_-ARs essentially trigger the G_s_/AC/cAMP/PKA pathway but are also involved in nonclassical G_i_ signaling displaying adverse effects on PKA activation and the inotropic response mediated by G_s_ [258].

There is increasing evidence that gender is highly related to cardiovascular states of health and disease. Whether androgens play a significant role in these dissimilarities is still investigated. Moreover, the genomic effects of T on ventricular cardiomyocytes' performance have been demonstrated. In this regard, Golden et al. showed that this androgen increased the mRNA expression of several critical Ca^2+^-handling proteins. Treatment of rat ventricular cardiomyocytes with T increased the levels of gene expression of L-VDCC, *β*_1_-AR, and NCX with 8 and 24 hours of exposure [259]. The T-induced changes in the mRNA expression levels of the mentioned proteins could be related to the improvement of the function of the cardiomyocytes and also be implicated in the development of hypertrophy and heart failure. These results point out an essential role of T in sex-related differences in the cardiac function.

Besides their electrophysiological properties, VDCCs are also classified using a standard nomenclature based on their molecular features of the pore-forming *α*1-subunit. Therefore, VDCCs are named using the chemical symbol of the permeating ion (Ca) with the physiological modulator (voltage) indicated as a subscript (Ca_V_). A numerical identifier resembles the channel *α*1-subunit gene subfamily (1 to 3) and the order of discovery of the *α*1-subunit within that subfamily (1 through *n*). According to this nomenclature, the L-VDCCs are represented by Ca_V_1.1–Ca_V_1.4 subunits, and T-VDCCs correspond to Ca_V_3.1–Ca_V_3.3 [260]. In this regard, it has been reported that chronic administration of T enhanced Ca^2+^ influx through L- and T-VDCCs due to an increased expression of the Ca_V_1.2, Ca_V_3.1, and Ca_V_3.2 subunits in ventricular cardiomyocytes, initially upgrading their performance but subsequently bringing the cell into new _b_[Ca^2+^]_i_ [261, 262]. These studies suggest that augmented _b_[Ca^2+^]_i_, via the upregulation of the Ca^2+^ channels aforementioned, might contribute to chronic cardiac pathogenesis when T levels are elevated.

While the nongenomic effects of T on the CM have been reported, the studies are scarce and seem to be contradictory regarding the Ca^2+^ handling and the cardiac contraction/relaxing outcomes. On the one hand, a group of researchers demonstrated in cultured rat cardiomyocytes that the acute exposure to T rapidly increased [Ca^2+^]_i_, and this augment was not abolished by an antagonist of the androgen receptor. Elegantly, the authors confirmed that the mechanism involved in the T-induced increase in [Ca^2+^]_i_ was mediated by the activation of a plasma membrane AR associated with a pertussis toxin- (PTX-) sensitive G protein (Gi/o) and with the activation of the PLC-IP_3_ signaling pathway leading to cardiac hypertrophy and failure [263]. The activation of PLC may be mediated through the action of *βγ*-subunits of the Gi/o proteins [264, 265]. On the other hand, it has been shown that acute exposure of T decreased the L- and T-VDCC activity by reducing their open probability [262, 266].

### 4.1. The Effects of Testosterone on Adrenergic Receptors in the Cardiac Muscle

Gender-related differences in cardiovascular disease (CVD) seem to be affected by age. It is well documented that the risk of dying for men between ages 45 and 64 from a CVD is higher than for women in the premenopausal period; however, there is a slight increase in the risk of CVD death in women after menopause [267].

The role of sex hormones in CVD is still unclear; particularly, a controversy about the effects of T in cardiovascular (CV) health and disease currently exists in the medical community. It is generally accepted that normal levels of T are beneficial for CV health in men, and a decline in these levels is related to an increase in CV events [268]. Nevertheless, a potential risk of developing CV events in patients receiving testosterone therapy has been reported [269, 270]. In postmenopausal women, endogenous elevated levels of total T (>0.9 nM) have been associated with CVD risk factors, such as high blood pressure and insulin resistance [271–275]. In this context, it has been postulated that estrogen augments the levels of sex hormone-binding globulin (SHBG). After menopause, the loss of ovary function leads to a general decline in sex steroid levels. Moreover, the fall of estrogen may lead to decreased levels of SHBG and higher free androgen levels [276]. Therefore, higher androgen levels and decreased estrogen levels in postmenopausal women have been suggested to be partially responsible for the CDV risk [268, 275]. Contrastingly, a study performed by Kaczmarek et al. demonstrated that low T levels are associated with coronary artery disease (CAD) in postmenopausal females [277]. During the menopausal transition, obesity is closely associated to CVD since it favors the secretion of proinflammatory cytokines, reactive oxygen species (ROS), and prothrombotic mediators [278–282]. Obesity promotes an unfavorable lipid profile which is associated with the development of CVD in elderly women [283, 284]. This profile is characterized by low high-density lipoprotein (HDL), higher total cholesterol (TC), and triacylglycerol (TAG) plasma levels [285]. In this regard, it was proved that oral DHEA therapy increased HDL and reduced TAG and LDL in adrenal-androgen-deficient postmenopausal women [286]. Also, obesity is a common feature of PCOS, exacerbating its symptoms and conferring a greater risk for CVD. The androgenic status, i.e., hyperandrogenism, needs to be considered when evaluating the metabolic and CV risk in PCOS women [200]. The ongoing controversy regarding the role of T in CVD might be moderately explained by the interaction between T and adrenoceptors in cardiac myocytes as discussed in the following.

It has been proposed that low plasmatic levels of T in older men are associated with an increase in atherosclerosis and cardiovascular risk, suggesting that this androgen plays a cardioprotective role against CVDs, such as coronary heart disease (CHD) and chronic heart failure (CHF) [287]. Moreover, it has been reported that an association between younger age at menopause and a greater risk of CDH in women coursing a natural menopause process [288]. Furthermore, T  could confer cardiac protection against ischemic injuries by increasing the effects of the *α*_1_-AR signaling pathway. The activation of *α*_1_-AR improves the myocardial performance after an infarction, reducing injury and arrhythmias [289]. CHD is characterized by myocardial ischemia and cardiac injury [290]. In this regard, it has been shown that patients with CHD have lower androgen levels than healthy men and that low doses of T  improved ischemic threshold in men suffering from angina [291, 292]. Furthermore, the administration of  T  enhanced the function recovery of the myocardium after a no-flow ischemia challenge in rats [293]. These observations point out to a reduction induced by T in the susceptibility to present myocardial ischemia and favor dilation of the coronary artery [294].

**Figure 4:**
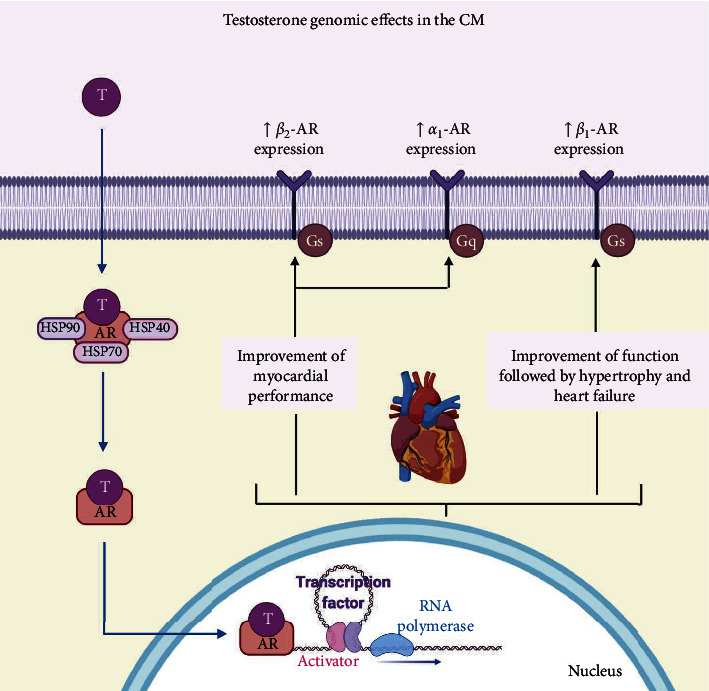
Genomic androgen effects on the adrenergic system in the cardiac muscle (CM). The genomic AR signaling involves androgen crossing the plasma membrane, entering the cytoplasm, dissociation of chaperone proteins, and binding to the AR. Testosterone (T) stimulates the expression of the *α*_1_-adrenergic receptor (*α*_1_-AR) and *β*_2_-adrenergic receptor (*β*_2_-AR), improving ionotropic activity and leading to the development of contraction without cardiotoxic effects. In addition, T increments the expression of the *β*1 adrenergic receptor (*β*1-AR), acutely improving the cardiomyocytes' function, but chronically leading to hypertrophy and heart failure.

The SNS (through norepinephrine) activates *α*- and *β*-ARs controlling the CM tone. However, during myocardial ischemia, the release of norepinephrine increases the risk and contributes to cardiac injury [295, 296]. In this regard, it has been shown that the T-induced overexpression of the *β*_1_-AR triggered proapoptotic pathways, weakening the cardiac structure and accelerating heart injury and failure progression [259, 297]. This overexpression also led to muscle hypertrophy in mice while producing an initial increase in contractility followed by a progressive dysfunction (Figure 4) [298].

On the contrary, the *α*_1_-ARs may play an important role in cardioprotection, specifically, the *α*_1A_-subtype. The overexpression of *α*_1A_-AR can improve the outcome after myocardial infarction [299], cardiac contractility, and reduced arrhythmias [119, 300]. In 2008, Tsang et al. demonstrated in rat ventricular myocytes that T replacement therapy (TRT) upregulated the *α*_1_-AR expression and augmented the cardiac responses, leading to a reduction in ischemia and cardiac injury [289]. Later on, in 2009, the same research group demonstrated that T enhanced the contractile function induced by the stimulation of both *α*_1_- and *β*_1_-AR in perfused rat hearts (Figure 4). Also, T treatment accelerated the relaxing response of the cardiac tissue. Interestingly, both phenomena were mediated by the AR [301]. The enhanced contractile response was explained since T augmented the function of RyR, leading to increased Ca^2+^ release from the SR. Otherwise, the augmented relaxing response was due to a more efficient activity of NCX regarding *α*_1_-AR stimulation and a heightened SERCA activity, accompanied with increased phosphorylation of PLB in the case of *β*_1_-AR stimulation [301]. Interestingly, they additionally found that the absence of T downregulated the expression of *β*_2_-AR in rat hearts, indicating that this androgen may also interact with this receptor subtype [289]. Moreover, it has been documented that the activation of *β*_2_-AR reduced apoptosis and increased the contractile mechanisms but did not accelerate relaxation as *α*_1_-AR and *β*_1_-AR stimulation did [302]. Although several studies have conducted about the relationship between cardiac function and androgens, more information is required to determine if T might play a key role in CHD.

Testosterone has also been associated with CHF [303]. This disease is a metabolic syndrome characterized by endocrine and inflammatory alterations, including elevated circulating catecholamine levels [304]. Testosterone deficiency (in hypogonadal subjects) has been demonstrated in 26% to 37% of male patients with CHF [305, 306]. Moreover, drugs used in CHF treatment, e.g., spironolactone and *β*-blockers, may diminish the function of Leydig cells, leading to a decline in the production of T [307, 308]. The low levels of T have been associated with reduced ejection fraction and increased systemic vascular resistance [309]. In this regard, the effects of T on *β*-AR have been investigated. In 2011, Sun et al. demonstrated that a TRT  in a heart failure rat model reversed the damage (decrease in contractility, apoptosis, and fibrosis in cardiomyocytes) through the protection of the cardiac *β*-adrenergic system. Notably, the stimulation of the AR by T upregulated the expression of *β*_2_-AR, improving the myocardial performance (Figure 4) [163]. Furthermore, it has been proposed that TRT in men with CHF induced an increase in the cardiac output and afterload [310].

The genomic regulation of the *β*-AR has been associated with cardiac remodeling and heart failure [311, 312]. In this regard, it has been shown that exercise training in rats reverses *β*-AR dysfunction by reducing the levels of G protein-coupled receptor kinase-2 (GRK2), an enzyme implicated in *β*_1_-AR and *β*_2_-AR dysregulation in CHF [313–315]. Moreover, exercise seems to restore the adrenal GRK2/*α*_2_-AR/catecholamine production axis [313]. Also, exercise augments vascular *β*-AR responsiveness and diminishes the activity of GRK2 [316]. Interestingly, *β*_1_-AR expression in the heart would be directly influenced by anabolic-androgenic steroids (AAS, synthetic derivatives of T) [317]. The use of AAS in combination with resistance training frequently improves the physical performance and helps athletes gain muscle mass and strength [318, 319]. However, numerous AAS abuse side effects include endocrine (hypogonadism) and detrimental cardiovascular issues [320–322]. For instance, vigorous training, anabolic steroid abuse, and the sympathetic nervous system's stimulation in mice increased cardiac levels of IL-1*β* and TNF-*α* and plasmatic levels of total cholesterol [320]. Furthermore, it has been demonstrated that the use of AAS induced cardiac hypertrophy and increased myocardial susceptibility to ischemia injury [322, 323]. In this context, the administration of nandrolone (AAS) to male rats under an exercise training protocol increased the expression of *β*_1_- and *β*_2_-AR in the cardiac right atrium, provoked the prolongation of the QTc interval, and increased the BP [324]. In addition, the exposure of nandrolone augmented hypertension in SHR rats and *β*_1_-AR protein expression in the left ventricle [317]. These data suggest that myocardial injury may be predisposed by high-performance training, steroid abuse, and the sympathetic nervous system's stimulation. Moreover, these insights may explain cardiac ailments and deaths in athletes under an AAS regimen.

Given the differences between studies showing the protective role of T in CV events and reports pointing out adverse CVD outcomes, it has been remarkably proposed that the use of **T**, as a treatment in CVD, should only be considered for male patients with a diagnosis of hypogonadism. Moreover, due to the increase of T therapy for postmenopausal women, the potential risk of developing CDV events needs further research [268, 275].

## 5. Conclusions

The adrenergic system plays a pivotal role in the control of vascular, airway, and cardiac physiology. A relationship between androgens with the adrenergic system of these tissues is proposed. This review summarizes that, in the vascular smooth muscle, T, via the androgen receptor, reduces the AC expression and increases the *α*_1_-AR expression, leading to high BP and hypertension. Moreover, in the airway smooth muscle, T, via nongenomic action, potentiates the *β*-adrenergic-induced relaxation through the inhibition of COMT or by the abolition of extraneuronal uptake. This androgen, via a genomic effect, also augments the expression of *β*_2_-AR and induces an increase in the relaxing responses to salbutamol. In the cardiac muscle, T upregulates the expression of *α*_1A_-AR and *β*_2_-AR mediated by the AR signaling, improving the myocardial performance. Moreover, T also increments *β*1-AR expression, improving the cardiomyocytes' function; however, the enhancement in muscle work during a long period ends up developing hypertrophy and heart failure.

Consequently, we might argue that androgen genomic actions have deleterious effects in the VSM favoring hypertension. Nevertheless, in the ASM, nongenomic and genomic actions of androgens contribute to diminish the hyperresponsiveness of this tissue, favoring the absence of asthma symptoms. Therefore, androgens could be a therapeutic alternative for asthma treatment. However, in heart diseases, further research is required to determine the possible therapeutic use of androgens in these ailments.

Finally, the use of T and DHEA as a therapeutic tool for the treatment of asthma symptoms or some cardiovascular diseases, is questionable. T has virilizing adverse effects, androgenic actions that favor prostate cancer, and its aromatization leads to the production of estrogens. Additionally, DHEA is further biotransformed into various sex steroids, such as T and estrogens, with their subsequent effects. However, 5*β*-DHT, a well-known T metabolite without genomic effects, could be a prospective therapeutic agent for the treatment of these illnesses.

## Data Availability

No data were used to support this study.

## Abbreviations

AAS: Anabolic-androgenic steroids
AC: Adenylyl cyclase
AC-6: Adenylyl cyclase 6
AHR: Airway hyperresponsiveness
*α*_1_-ARs: Alpha-1-adrenergic receptors
*α*_2_-ARs: Alpha-2-adrenergic receptors
ANF: Atrial natriuretic factor
AR: Androgen receptor
ASM: Airway smooth muscle
Basic bFGF: Fibroblast growth factor
*β*-ARs: Beta-adrenergic receptors
BK_Ca_: Ca^2+^-activated K^+^ channels of high conductance
BP: Blood pressure
cAMP: 3′, 5′-Cyclic adenosine monophosphate
cGMP: 3′, 5′-Cyclic guanosine monophosphate
CGRP: Calcitonin gene-related peptide
CHD: Coronary heart disease
CHF: Chronic heart failure
CIRC: Ca^2+^-induced Ca^2+^ release
CM: Cardiac muscle
COMT: Catechol-O-methyl transferase
COPD: Chronic obstructive pulmonary disease
CV: Cardiovascular
CVD: Cardiovascular disease
DAG: Diacylglycerol
DHEA: Dehydroepiandrosterone
5*α*-DHT: 5*α*-Dihydrotestosterone
5*β*-DHT: 5*β*-Dihydrotestosterone
EGFR: Epidermal growth factor receptor
ERK1/2: Extracellular signal-regulated kinases 1 and 2
FSH: Follicle-stimulating hormone
GDP: Guanosine diphosphate
GPRC6A: G protein-coupled receptor family C group 6-member A
GPCR-_q_*α*: GPCRs coupled to the _q_*α* subunit
GPCRs: G protein-coupled receptors
GPCR-_s_*α*: GPCRs coupled to the _s_*α* subunit
GRK2: G protein-coupled receptor kinase-2
GPs: Ganglionic plexuses
GTP: Guanosine-5′-triphosphate
HA: Hyperandrogenism
HDL: High-density lipoprotein
HGFR: Hepatocyte growth factor receptor
3*β*-HSD: 3*β*-Hydroxysteroid dehydrogenase
17*β*-HSD: 17*β*-Hydroxysteroid dehydrogenase
ICNS: Intrinsic cardiac nervous system
I_K_: K^+^ currents
IK_Ca_: Ca^2+^-activated K^+^ channels of intermediate conductance
IL-13: Interleukin-13
IL-17: Interleukin 17
IP_3_: Inositol-1, 4, 5-triphosphate
_b_[Ca^2+^]_i_: Intracellular basal Ca^2+^ concentration
[Ca^2+^]_i_: Intracellular Ca^2+^ concentration
K_ATP_: ATP-sensitive K^+^ channels;
K_Ca_: Ca^2+^-activated K^+^ channels
K_IR_: Inward-rectifier K^+^ channels
K_V_: Voltage-dependent delayed rectifier K^+^ channels
K_V_1.5: Voltage-dependent delayed rectifier K^+^ channel 1.5
LH: Luteinizing hormone
L-VDCCs: L-type voltage-dependent Ca^2+^ channels
MAPKs: Mitogen-activated protein kinases
MEK: Mitogen-activated protein kinase kinase
MLCK: Myosin light-chain kinase
NCX: Na^+^-Ca^2+^ exchanger
NCX_REV_: Reverse-mode Na^+^/Ca^2+^ exchanger
NO: Nitric oxide
NPY: Neuropeptide Y
PCa: Prostate cancer
PCOS: Polycystic ovary syndrome
PDGF: Platelet-derived growth factor
PGE_2_: Prostaglandin E_2_
PI3K: Phosphatidylinositol 3-kinase
PKA: Protein kinase A
PKC: Protein kinase C
PKG: Protein kinase G
PLB: Phospholamban
PLC: Phospholipase C
PLC_*β*_: Phospholipase C_*β*_
PMCA: Plasma membrane Ca^2+^-ATPase
PNS: Parasympathetic nervous system
PTX: Pertussis toxin
ROCCs: Receptor-operated Ca^2+^ channels
RyR_2_: RyR isoform 2
RyRs: Ryanodine receptors
SERCA: Sarcoplasmic reticulum Ca^2+^-ATPase
5-HT: Serotonin
SHR: Spontaneously hypertensive rats
SK_Ca_: Ca^2+^-activated K^+^ channels of low conductance
SNS: Sympathetic nervous system
SOCCs: Store-operated Ca^2+^ channels
SR: Sarcoplasmic reticulum
T: Testosterone
TAG: Triacylglycerol
TC: Total cholesterol
TNF-*α*: Tumor necrosis factor alpha
TRPC: Transient receptor potential channels
TRPC3: Transient receptor potential canonical 3
TRT: Testosterone replacement therapy
T-VDCCs: T-type voltage-dependent Ca^2+^ channels
VDCCs: Voltage-dependent Ca^2+^ channels
VEGFR-1/2: Vascular endothelial receptors 1/2
VSM: Vascular smooth muscle
ZIP9: Zinc-regulated transporter [Zrt]-protein 9.
